# Contrasting effects of nitrogen and phosphorus additions on soil nitrous oxide fluxes and enzyme activities in an alpine wetland of the Tibetan Plateau

**DOI:** 10.1371/journal.pone.0216244

**Published:** 2019-05-02

**Authors:** Yunyun Zhang, Chunmei Wang, Yun Li

**Affiliations:** College of Environmental Science and Engineering, Beijing Forestry University, Beijing, China; Tennessee State University, UNITED STATES

## Abstract

Alpine wetlands are important ecosystems, but an increased availability of soil nutrients may affect their soil nitrous oxide (N_2_O) fluxes and key enzyme activities. We undertook a 3-year experiment of observing nitrogen (N) and/or phosphorus (P) addition to alpine wetland soils of the Tibetan Plateau, China, with measurements made of soil extracellular enzyme activities and soil N_2_O fluxes. Our study showed that soil N_2_O flux was significantly increased by 72% and 102% following N and N+P additions, respectively. N addition significantly increased acid phosphatase (AP) and β-1, 4-*N*-acetyl-glucosaminidase (NAG) activities by 32% and 26%, respectively. P addition alone exerted a neutral effect on soil AP activities, while increasing NAG activities. We inferred that microbes produce enzymes based on ‘resource allocation theory’, but that a series of constitutive enzymes or the treatment duration interfere with this response. Our findings suggest that N addition increases N- and P-cycling enzyme activities and soil N_2_O flux, whereas P addition exerts a neutral effect on P-cycling enzyme activities and N_2_O flux after 3 years of nutrient applications to an alpine wetland.

## Introduction

Nitrous oxide (N_2_O) is the main contributor to global warming. The gas has a warming potential that is 298 and 21 times as high as that of carbon dioxide and methane, respectively, and adds greatly to the greenhouse effect and ozone depletion in the stratosphere [1]. N_2_O emissions from terrestrial soils have been determined to be the most important source of atmospheric N_2_O flux (57%) [2], which increased by nearly 20% between the 1750s and 2011s [3]. Currently, there is a large body of evidence to show that global atmospheric nitrogen (N) deposition has increased dramatically [4], with the average bulk deposition of N having increased from 13.2 kg N ha^−1^ yr^−1^ to 21.1 kg N ha^−1^ yr^−1^ between the 1980s and 2000s [5]. Chronic deposition of N into the soil could contribute a substantial proportion of the available N and affect the microbial processes of nitrification and denitrification that are the main systems by which N_2_O is produced in soils. In many terrestrial ecosystems, N is a major limiting nutrient and coupling occurs between nutrient cycles with interactions between soil N and phosphorus (P) possibly influencing the biogeochemical responses to an excess of one nutrient in a specific stoichiometric ratio [6–8]. For instance, an excess of N could influence available pools of P by virtue of shifting enzyme activities, mineralization and uptake processes, thereby having a strong influence on N_2_O emissions from soils [2, 6].

A meta-analysis showed that N_2_O emissions from N addition plots (0–400 kg N ha^−1^ yr^−1^) were 134% higher than that of control plots in terrestrial ecosystems as a consequence of increasing available mineral N in soils [9]. However, several experimental studies have found N_2_O flux had negative response to P addition in plantation soils [10–12], with one suggested mechanism being that P addition simulated plant uptake of soil N, and reduced the available N substrate; whereas, the combined application of P and N had a neutral effect on N_2_O flux, although P addition reduced the stimulation of N_2_O emission by N addition in P-limited forest soil [11, 13, 14]. In contrast, other studies have demonstrated that the combined application of P and N significantly increased N_2_O flux in forest soil. This may be due to increased microorganism abundance and activity in soils driven by the synergistic effect of N and P [2, 6, 15]. Hence, terrestrial ecosystems can respond differently to increasing levels of N and P addition and N_2_O flux can increase or decrease due to differences in the ecosystem and soil properties.

Soil enzymes involved in biochemical processes are closely associated with nutrient cycling and energy conversion [16]. Bai et al. [17] showed urease activity could reflect N_2_O flux, which was not only affected by environmental factors. A significant negative correlation between acid phosphatase (AP) and N_2_O flux was found under different soil type [18]. In nutrient-deficiency soil environment, microbes hydrolyze complex organic matter via extracellular enzymes and release C, N and P, which are absorbed and utilized by microbes and plants [19]. This is consistent with the economic theories of microbial metabolism indicating that if nutrients are limited, enzyme production increases, whereas under nutrients-rich conditions, enzyme production decreases [20]. For example, in P-restricted soil, phosphatase activity increased [21], however, the application of P fertilizers increased the availability of P in the soil, and phosphatase activities were suppressed [22]. Enzyme activities in the soil can be used as an indicator of the nutritional requirements of microbes and plants [22]. Nevertheless, according to previous studies, N and/or P addition showed positive, negative or neutral effects on soil enzyme activities [2, 15, 21], depending on the particular soil properties, the levels of N and/or P additions, and the enzymes assayed.

Although the effect of N and P addition on the processes of nutrient recycling has been studied in many ecosystems, the results of research on interactions between soil N and P and how they influence biogeochemical responses to enrichment by one nutrient are inconsistent [9, 10–14]. In particular, how N and P addition influence N_2_O flux and enzyme activities are not well understood in the alpine wetland of the Tibetan Plateau. The plateau covers nearly one-quarter of China’s land area, which regarded as "the roof of the world" and "the third pole". This area has the highest altitude in the world and is one of the most sensitive regions to global atmospheric changes [23]. Crucially, the annual average temperature of the alpine wetland is about 0°C, which results in a low rate of N and P decomposition. In addition, the alpine wetland receives atmospheric N deposition rate to a value of 10–15 kg N ha^−1^ yr^−1^ [24]; also, the region is one of the most important animal husbandry area in China, which produces a large amount of livestock manure and increases N and P input into wetland soils [25]. Therefore, a field-based *in situ* controlled experiment carried out to investigate the response of soil N_2_O flux and enzyme activities to N and P addition in an alpine wetland ecosystem. Specifically, plant biomass, soil N availability, N_2_O flux, soil microbial biomass, and several soil extracellular enzyme activities related to N-, and P-cycling were measured as response variables during the growing season from 2014 to 2016. Many terrestrial ecosystems are N limited and the utilization of N and P is in accordance with a certain stoichiometric ratio. Hence, we hypothesized that (1) N and/or P application could increase N_2_O flux, with N and P promoting N_2_O flux more than either nutrient application alone; (2) N addition would have a positive effect on P-cycling enzyme activities but inhibit the those of N-cycling enzymes, while P addition would show the opposite pattern according to the theory of resource allocation [20].

## Materials and methods

### Site design

This study was conducted in an alpine wetland in the Zoigê National Reserve (102°56'59" E, 33°34'54" N, at 3452 m a.s.l.), located in the eastern region of the Tibetan Plateau, China. The mean annual temperature and precipitation in this area is approximately 1.4°C (min: –10.6°C in January, max: 10.8°C in July) and 700 mm, respectively [26]. Precipitation, with seasonal variation, occurs mainly between April and October; the soil water content averages 132%. The soil here is peaty, acidic and rich in organic C, with an average peat layer thickness of 2–5 m [27]. *Carex muliensis* and *C*. *lasiocarpa* are dominant plant species in the alpine wetland.

Before N and P were added, soil samples from a depth of 0–15 cm were collected (with four replicates) in each plot using corers with a diameter of 3.5 cm in May 2014. Soil organic C and total N contents were ~26% and ~1%, respectively, and soil C: N ratios ranged from 21 to 22 (Table 1). The initial soil properties showed no significant differences among the treatment plots.

**Table 1:** Initial physical-chemical properties of the study site among different N and/or P addition (n = 4).

Variable	Control	N	P	N+P
**pH**	6.16±0.10_a_	6.38±0.18_a_	6.27±0.09_a_	6.18±0.13_a_
**Organic C (g kg**^**−1**^**)**	260.64±2.58_a_	268.37±3.62_a_	260.03±3.39_a_	258.28±2.97_a_
**Total N (g kg**^**−1**^**)**	12.03±0.12 _a_	12.11±0.11 _a_	12.05±0.12 _a_	12.07±0.13 _a_
**C: N**	21.66±0.21_a_	22.16±0.18_a_	21.57±0.17_a_	21.37±0.20_a_
**NH**_**4**_^**+**^**-N (mg kg**^**−1**^**)**	16.86±0.34_a_	17.10±0.27_a_	16.26±0.32_a_	17.25±0.31_a_
**NO**_**3**_^**-**^**-N (mg kg**^**−1**^**)**	9.31±0.34_a_	9.49±0.29_a_	8.93±0.31_a_	9.53±0.3_a_
**Available P (mg kg**^**−1**^**)**	8.14±0.24_a_	8.05±0.21_a_	8.25±0.25_a_	8.02±0.23_a_

Date are expressed as means ± SE. Means with different lowercase letters in the same row are significantly different at *P* < 0.05. N: NH_4_NO_3_ addition treatment; P: NaH_2_PO_4_ addition treatment; N+P: NH_4_NO_3_ and NaH_2_PO_4_ co-addition treatment.

### Experimental treatment

The plots in this study were randomly distributed on flat wetland ground. In total, we established 16 plots, each 10 m × 10 m, within the experimental site in 2014. Buffer zones were 5-m wide between the plots. N was uniformly applied to soil at the rate of 0 or 20 kg N ha^−1^ yr^−1^ in the form of NH_4_NO_3_. Each treatment of N was accompanied with 0 or 10 kg P ha^−1^ yr^−1^ as NaH_2_PO_4_. There were four treatments: control, N addition, P addition, and N+P addition. Each treatment had four replicates. We chose a higher than natural N and P addition rate, which is typical of that normally applied to wetland soils on the Tibetan Plateau [28–30].

Over a 3-year period, N and/or P were added equally on the first day of the month during the growing season from 2014 to 2016 (i.e., from May to September). Additions of NH_4_NO_3_ and NaH_2_PO_4_ were dissolved in 7.5 L of deionized water and sprayed evenly on the soil surface with a sprayer and the same amount of deionized water was sprayed on the control plots to reduce the impact of water on the plots.

### N_2_O flux sampling

Soil N_2_O flux was measured using a static opaque chamber equipped with a stainless steel base and a square top. During the entire experiment, a stainless steel base was embedded into a soil area selected as the experimental plot. A removable top (50 cm × 50 cm × 50 cm) containing two small fans ensuring proper mixing was installed inside the fixed base to collect gas. Air temperatures (Ta) and soil temperatures (Ts) at a depth of 5 cm were recorded using a digital thermometer. Gas was collected four times, at 09:00–11:00 a.m. on the 7^th^, 14^th^, 21^st^ and 28^th^ day after N and P addition in each month from May to September (2014–2016). At each time point, four 100-mL gas samples were collected from each plot at 10 min intervals using plastic syringes. Gas sampling lasted for half an hour on each plot and samples were stored within 12 h, before being measured in the gas chromatograph (Agilent 7890A, Agilent Technologies Inc., Palo Alto, CA, USA.).

Soil N_2_O flux (μg N m^−2^ h^−1^) was calculated from 2014 to 2016 as follows [31]:

$$F=\frac{dc}{dt}\times D\times H\times 1000$$ (1)

where F is soil N_2_O flux (μg N m^−2^ h^−1^); dc/dt is the rate of change between time and N_2_O concentration (10^−6^ min^−1^); D refers to molar density of air (mol m^−3^); $D=\frac{MP}{RT}$; M is the molar mass of N_2_O-N (g mol^−1^); R is gas constant (J mol^−1^ K^−1^); P and T is air pressure (Pa) and air temperature (K) inside the chamber, respectively, and H is the height of the sampling box (m).

Soil cumulative N_2_O flux (kg N ha^−1^ yr^−1^) was calculated spanning the growing season following the method described by Xu et al. [31].

$$Cumulative\;N_{2}O\;flux=\frac{\sum_{i=1}^{n}0.5\times (F_{i}+F_{i+1})\times (t_{i+1}-t_{i})\times 24}{100000}$$ (2)

where F is the N_2_O flux (μg N m^−2^ h^−1^); *i* is the sampling number; i.e., samples collected on the 7^th^ in May as 1 and those collected next on the 28^th^ in September as 20; t is the sampling interval time based on the Julian day (day).

### Soil samples and their analyses

Surface litter was first removed and four soil cores (3.5 cm in diameter) from the topsoil (0–15 cm) were randomly collected on four occasions. The soil samples were stored at –4°C before being measured. Soil water content and bulk density were measured using the oven-drying method (at 105°C, for 8 h) and core method, respectively. The water-filled pore space (WFPS, %) of soil was calculated as described by Jian et al. [18].

$$WFPS=\frac{water\;content\times bulk\;density\times 100}{1-bulk\;density/2.65}$$ (3)

Soil pH was measured on a soil-water suspension (1: 2.5) using pH meter (PB-10 pH meter, Sartorius Co.). The NH_4_^+^-N and NO_3_^−^-N concentrations were measured by extraction with KCl and then followed by colorimetric analysis on a spectrophotometry (T6-1650E UV-Vis spectrophotometer, Purkinje Co., China) and dual-wavelength spectrophotometry (T6-1650E UV-Vis spectrophotometer, Purkinje Co., China), respectively. Available P was extracted with sodium bicarbonate solution and analyzed by colorimetry [11]. Soil microbial biomass was determined using a chloroform fumigation extraction method [32]. The enzyme activities of AP and β-1, 4-*N*-acetyl-glucosaminidase (NAG) were determined using 96-well microplates as described by Turner et al. [21]. In brief, we homogenized 1.00 g soil in 125 ml of 50 mM Tris buffer (pH 7). Soil suspension then was added to a 96-well microplate along with 50 ml of 200 mM fluorogenic substrate in each well of the measurement plants. Measurement plates incubated for 6 h at 25°C in the dark were measured with a fluorescence spectrometer (Spectramax M2, Molecular Devices, Sunnyvale, CA, USA.).

### Plant biomass analysis

Aboveground biomass was estimated by clipping the live biomass of vegetation in the plots. Specifically, the arial parts of living plant were harvested from 25 cm × 25 cm quadrat randomly established in each plot at the end of the growing seasons in 2014, 2015 and 2016. The ensuing dry matter weighed as aboveground biomass after being oven-dried at 70°C for 48 h. A part of plant was used for chemical analyses after determination of dry mass. Total N of plants was determined by using the Kjeldahl method after wet digestion with sulfuric acid [33]. Four soil cores (3.5 cm in diameter) were sampled from the same quadrats at a depth of 0–15 cm to analyze soil root biomass. Root samples were cleaned with water and then oven-dried and weighed as belowground biomass. After completing these measurements, all remaining harvested biomass was returned to the original quadrats in a random distribution.

### Statistical analysis

SPSS 22.0 software (IBM Corp., Armonk, NY, USA.) was used for statistical analyses. At *P* < 0.05, statistical tests were considered to be significant. Mean differences in soil properties, plant primary productivity, enzyme activities, soil microbial biomass C (MBC) and microbial biomass N (MBN), between different N and P additions (four treatments) were examined using one-way analysis of variance. Repeated-measures analysis of variance was used to analyze the effects of time, fertilizer addition (N and/or P addition), and their interactions on soil N_2_O flux and cumulative N_2_O flux. Pearson correlations were used to analyze the associations between soil properties and soil N_2_O flux, as well as AP and NAG. Linear regressions were performed to analyze the relationships between increasing N_2_O flux and increasing inorganic N concentrations from N and/or P additions (in comparison with control).

## Results

### Soil water conditions, temperature and plant biomass

The mean values of Ts and WFPS for the growing season were 16.55°C and 81%, respectively. However, N and/or P addition neither affected Ts nor WFPS (*P* > 0.05). Total biomass was 1065.32 ± 34.83 g m^−2^ in the control plot. There were increases in total biomass of 48% and 62% in the N and N+P addition via increases in both above- and underground biomass. However, the P addition only had a significant effect with respect to increased aboveground biomass (*P* < 0.05, Table 2).

**Table 2:** Effects of N and/or P addition on soil properties and plant biomass during the growing season from 2014 to 2016.

	Inorganic N (mg kg^−1^)	Available P (mg kg^−1^)	WFPS (%)	Ts (°C)	Aboveground biomass (g m^−2^)	Belowground biomass (g m^−2^)	Total biomass (g m^−2^)
**Control**	27.31±0.56_b_	9.02±0.02_b_	80±4_a_	16.32±0.27_a_	397.55±12.01_c_	667.77±32.63_b_	1065.32±34.83_b_
**N**	38.69±0.70_a_	8.28±0.02_c_	82±3_a_	16.56±0.25_a_	556.48±14.37_a_	1022.47±51.12_a_	1578.95±42.24_a_
**P**	23.83±0.43_c_	10.39±0.03_a_	81±3_a_	16.66±0.26_a_	477.75±12.73_b_	606.89±39.97_b_	1084.64±32.15_b_
**N+P**	37.55±0.69_a_	10.36±0.03_a_	81±4_a_	16.67±0.28_a_	607.30±13.14_a_	1120.51±50.32_a_	1727.81±48.66_a_

Date are expressed as means ± SE. Means with different lowercase letters in the same column are significantly different at *P* < 0.05. N: 20 kg N ha^−1^ yr^−1^; P: 10 kg P ha^−1^ yr^−1^; N+P: 20 kg N ha^−1^ yr^−1^ and 10 kg P ha^−1^ yr^−1^.

### N_2_O flux

**Fig 1:**
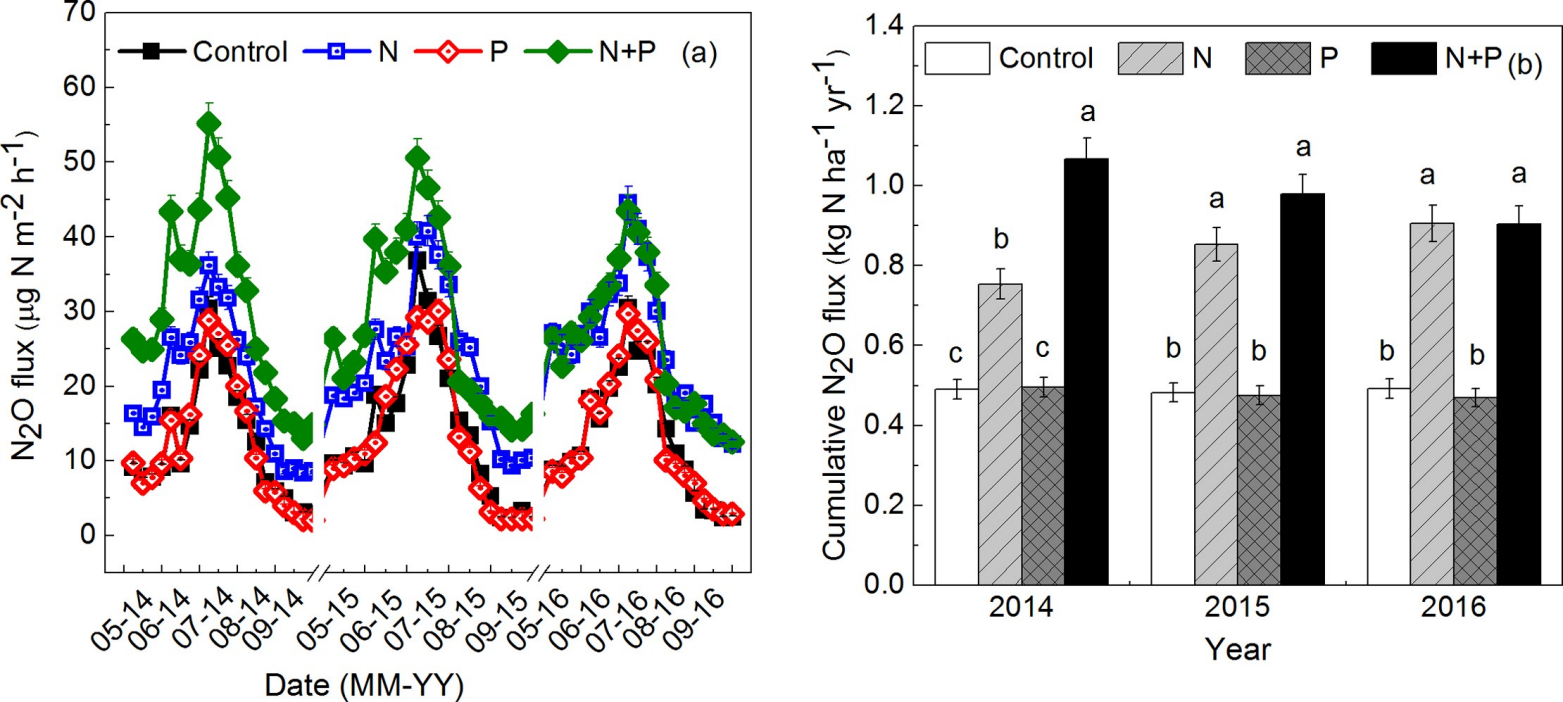
**N**_**2**_**O fluxes (a) and cumulative N**_**2**_**O flux (b) during the growing season from 2014 to 2016 among different N and/or P addition plots.** Vertical bars represent ± SE. Means with different lowercase letters in the same column are significantly different at *P* < 0.05. N: 20 kg N ha^−1^ yr^−1^; P: 10 kg P ha^−1^ yr^−1^; N+P: 20 kg N ha^−1^ yr^−1^ and 10 kg P ha^−1^ yr^−1^.

Soil N_2_O flux showed temporal fluctuations during the growing season (Fig 1A**)**. The peak in N_2_O flux values occurred in July, when it ranged from 27.66 ± 0.52 to 53.20 ± 0.76 μg N m^−2^ h^−1^, and the lowest values occurred in September (2.03 ± 0.04 to 15.68 ± 0.27 μg N m^−2^ h^−1^). In one month, soil N_2_O flux decreased first and then increased before July and after that the N_2_O flux gradually decreased after N and N+P additions. N_2_O flux increased significantly from an average of 13.55 ± 0.23 to 23.26 ± 0.34 μg N m^−2^ h^−1^ with the N addition, to 27.30 ± 0.42 μg N m^−2^ h^−1^ with N+P addition. However, N_2_O flux in the P addition plot was close to the N_2_O flux in the control plot (Fig 1A). The relationship between time (month) and fertilizer addition forms (N and/or P) had a significant effect on soil N_2_O flux (*P* < 0.05, Table 3).

**Table 3:** Repeated measures ANOVA on the effects of time, N and/or P addition form, and their interactions on soil N_2_O flux and cumulative N_2_O flux.

N_2_O flux				Cumulative N_2_O flux			
Subjects	d.f.	*F*	*P*	Subjects	d.f.	*F*	*P*
Between subject				Between subject			
Month	14	106.388	**0.001**	Year	2	0.044	0.957
Addition forms	3	192.080	**0.001**	Addition forms	3	107.632	**0.001**
Month × Addition forms	42	2.877	**0.001**	Year × Addition forms	6	2.361	0.062

N: 20 kg N ha^−1^ yr^−1^; P: 10 kg P ha^−1^ yr^−1^; N+P: 20 kg N ha^−1^ yr^−1^ and 10 kg P ha^−1^ yr^−1^.

Considering the cumulative N_2_O flux, it was significantly increased by N addition and N+P addition, whereas P addition had no significant effect on cumulative N_2_O flux. Cumulative N_2_O flux in N and N+P additions plots in comparison with control, increased by an average of 72% and 102% during the growing season from 2014 to 2016, respectively. However, the effect of N+P addition on the cumulative N_2_O flux was stronger than N addition alone in the first year (2014), but this difference became insignificant in the next two years (Fig 1B). Cumulative N_2_O flux was significantly affected by fertilizer addition forms, and there was no influence between year and fertilizer addition forms (Table 3).

### Response of soil microbes to N and/or P addition

Soil microbial biomass tended to significantly increased by N and N+P addition, but for P addition, it was close to the soil microbial biomass found in the control plot. In the N and N+P addition plots, MBC increased by an average of 44% and 47%, while MBN increased 52% and 56%, respectively (Table 4). There was no significant difference between N addition alone and N+P addition.

**Table 4:** Effects of N and/or P addition on acid phosphatase (AP) activities, β-1, 4-*N*-acetyl-glucosaminnidase (NAG) activities and soil microbial biomass C and N (MBC and MBN) during the growing season from 2014 to 2016 (n = 60).

	MBC (mg kg^−1^)	Increase from control	MBN (mg kg^−1^)	Increase from control	AP activities (nmol g^−1^ h^−1^)	Increase from control	NAG activities (nmol g^−1^ h^−1^)	Increase from control
**Control**	730.26±15.67_b_	-	135.55±2.85_b_	-	0.87±0.02_b_	-	6.52±0.15_c_	-
**N**	1050.46±20.46_a_	44%	206.38±4.18_a_	52%	1.15±0.03_a_	32%	8.19±0.17_b_	26%
**P**	747.37±15.58_b_	2%	141.61±3.02_b_	4%	0.85±0.02_b_	-2%	8.65±0.19_a_	33%
**N+P**	1075.75±21.19_a_	47%	210.86±4.62_a_	56%	0.89±0.02_b_	2%	8.27±0.17_b_	27%

Date are expressed as means ± SE. Means with different lowercase letters in the same column are significantly different at *P* < 0.05. N: 20 kg N ha^−1^ yr^−1^; P: 10 kg P ha^−1^ yr^−1^; N+P: 20 kg N ha^−1^ yr^−1^ and 10 kg P ha^−1^ yr^−1^.

AP activities only increased with N addition, from 0.87 ± 0.02 to 1.15 ± 0.03 nmol g^−1^ h^−1^ (32% increase). In contrast, the P addition and N+P addition apparently had negligible effects on AP activities during the study period. NAG activities under N addition, P addition and N+P addition were annually increased by an average of 26%, 33% and 27% over the control, respectively (Table 4).

### Soil properties and their correlation with N_2_O flux and enzyme activities

**Fig 2:**
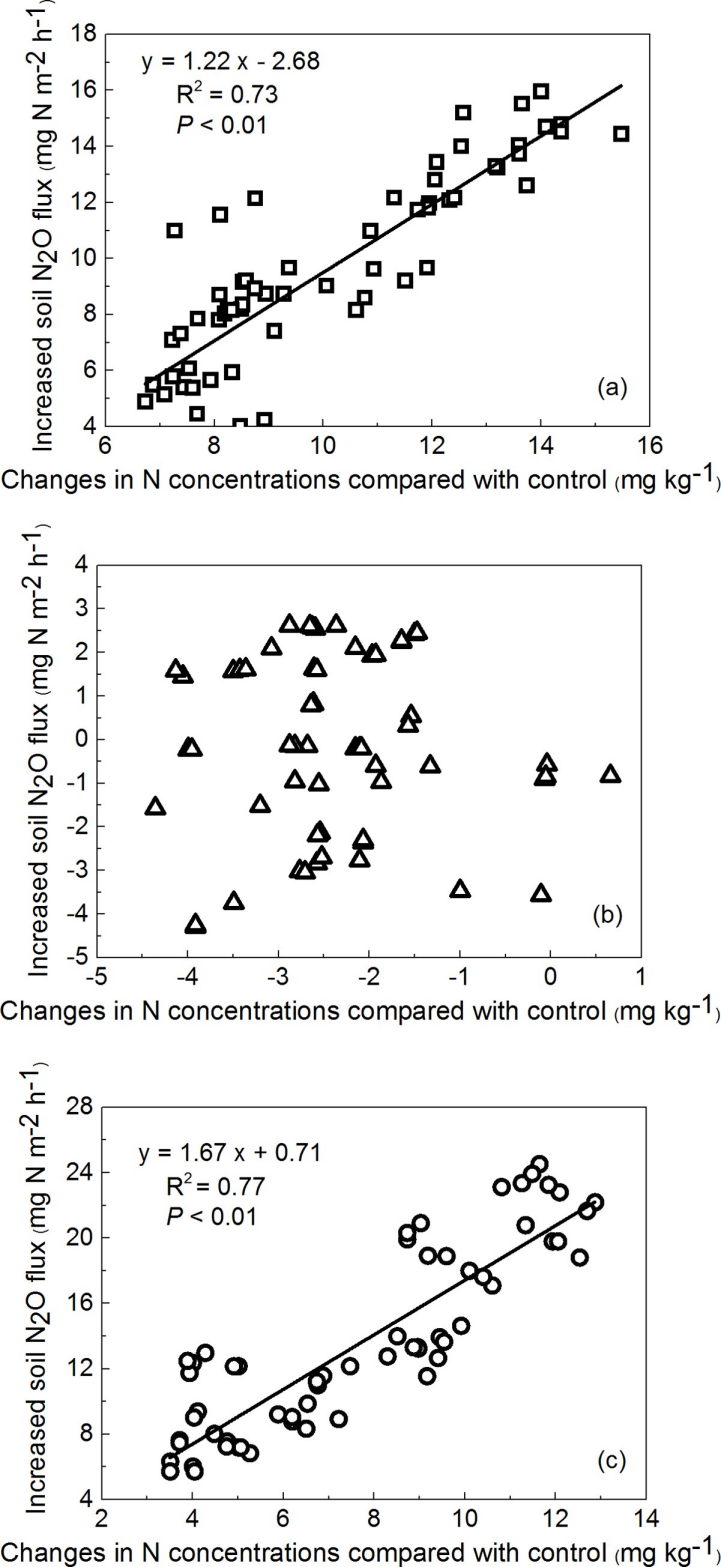
**Relationships between increased soil N**_**2**_**O flux and increasing inorganic N concentration applied with N (a), P (b) and N+P (c) addition compared with the control (n = 60).** N: 20 kg N ha^−1^ yr^−1^; P: 10 kg P ha^−1^ yr^−1^, N+P: 20 kg N ha^−1^ yr^−1^ and 10 kg P ha^−1^ yr^−1^.

Simple linear regressions revealed a positive relationship between increasing soil N_2_O flux and inorganic N concentration under N and N+P addition treatments in comparison with control (Fig 2). However, no such relationship was found between increased soil N_2_O flux and increased inorganic N concentration for the P addition plots.

The correlations showed that soil N_2_O flux, AP and NAG were positively associated with several key soil properties, including NH_4_^+^-N, NO_3_^−^-N, MBC, and MBN (Table 5). The strongest correlations occurred between soil N_2_O flux and MBN, while those between soil N_2_O flux and soil properties were stronger than those with AP or NAG and soil properties (except for Ts and available P). The correlations analysis showed that soil N_2_O flux were positively associated with NAG, while it was insignificant between soil N_2_O flux and AP (S1 Table).

**Table 5:** Correlation coefficients between soil acid phosphatase (AP), β-1, 4-*N*-acetyl-glucosaminnidase (NAG), N_2_O flux and soil properties.

Index	WFPS	Ts	available P	NH_4_^+^-N	NO_3_^−^-N	Microbial biomass C	Microbial biomass N
**N**_**2**_**O flux**	0.634**	0.541**	0.091	0.579**	0.802**	0.833**	0.880**
**AP**	0.308*	0.161	-0.247	0.488**	0.633**	0.513**	0.572**
**NAG**	0.553**	0.543**	0.035	0.533**	0.688**	0.630**	0.839**

Pearson correlation coefficients (r) and their significance (*P*) are given as

* *P* < 0.05 and

**P < 0.01, respectively.

## Discussion

### Promoting effects of N addition

Relatively low levels N or P (20 kg N ha^−1^ yr^−1^ or 10 kg P ha^−1^ yr^−1^) were added to moderately increase nutrient availability and minimize the impact on the ecosystem [6]. Soil N_2_O flux decreased first and then increased after N and N+P additions in one month before July, which may be due to declined available N in soil and increasing soil temperature (S1 Fig). After July, the N_2_O flux gradually decreased in one month with the decline of soil N availability and soil temperature.

Our results showed that the mean N_2_O flux in the control plot was 13.55 μg N m^–2^ h^–1^, which was comparable to the value (-2.05 to 110 μg N m^–2^ h^–1^) reported by Chen et al. [34] from different wetland soils on the Qinghai-Tibetan Plateau. Higher water content can lead to the anoxic environment in wetland soils, which facilitates the denitrification processes, producing and releasing large amounts of N_2_O into the atmosphere [35]. In our study area, soil N_2_O flux was increased by 72%, on average, under N addition treatments (20 kg N ha^−1^ yr^−1^) compared to the control. This value was lower than that found in a meta-analysis, which showed that N_2_O flux from N addition plots (10–562 kg N ha^−1^ yr^−1^) significantly increased by 216% in wetland ecosystems [36], also lower than the average increase of 134% in terrestrial ecosystems [9], indicating that N_2_O emission was significantly influenced by temperature, soil properties, ecosystem type and the amount of N fertilizer applied to the area. In our study, both above- and underground biomass was highly responsive (average 40% and 53% increase, respectively) to N additions, showing that N was the principal limiting nutrient in our study area. Therefore, N addition increased the substrate (NH_4_^+^-N and NO_3_^−^-N) for nitrification and denitrification processes and relieved the limitation of N in plants and microbes, increased enzyme activities (NAG activity) related to soil N recycling and increased N_2_O flux [7, 31, 37, 38].

### Neutral effect of P addition

In our research, no detectable change in N_2_O flux was found following P addition in the alpine wetland, thus rejecting our hypothesis that P addition causes an increase in N_2_O flux. This finding is consistent with work conducted in forest [2, 39] and grassland soil [40]. However, a negative response of N_2_O flux to P addition was also found in the field and laboratory, and the main explanation by which P reduced N_2_O flux was higher P availability after P addition enhanced N uptake by plants, and decreased the N available to nitrifying and denitrifying bacteria in soil, therefore reduced gaseous N losses [12, 41]. One nutrient addition could change the available pools of another by virtue of influencing uptake processes, mineralization and enzyme activities based on the theory of resource allocation, thereby having an influence on N_2_O emissions from soils [6]. Our study found that P addition decreased inorganic N concentration (average 13% decrease) in the soil, whereas aboveground biomass was significantly increased and the N content in biomass increased 20% (Table 2). We also observed that microbial biomass (MBC and MBN) was not significantly affected after P addition, while N-cycling enzyme activities (NAG) increased (Table 4). P additions may stimulate N cycling, while decreasing available pools of inorganic N in soil by promoting uptake of N in plants (Table 2), so that P addition exerted a neutral effect on N_2_O flux.

### Synergy of N+P addition

Although the P addition did not affect soil N_2_O flux, it was substantially increased by an annual average of 102% in the N+P addition plots in the studied alpine wetland. Moreover, the response of cumulative N_2_O flux to N+P addition was higher than N addition alone in the first year, which may be partly attributable to a synergic effect from the N+P addition, which is consistent with previous studies [2, 15]. In our study area, N+P addition could relieve N limitation of plants and microorganisms, and therefore increased plant and microbial biomass (Tables 2 and 4). Microbial growth used the available nutrient elemental in stoichiometric proportion. Soil inorganic N concentration and available P in N+P addition plot in comparison with control, increased by an average of 37% and 15%, respectively (Table 2). Synchronously adding N+P may have provided microorganisms with an adequate balanced element supply, thus augmenting the rates of nitrification and denitrification and increasing the N_2_O flux from soil [42, 43]. Besides, AP activities were decreased by an average of 23% in the N+P addition plots in comparison with N addition (Table 4). Phosphatase production requires a high cost of N [6], thus resulting in a transient increase in soil N availability under N+P addition treatments in comparison with N addition in the first year (40.56 vs 37.42 mg kg^−1^). The increase in soil N availability induced by N+P addition could stimulate nitrification and denitrifying bacteria, thereby enhancing N_2_O emissions without substrate N limitation. Hence, the effect of N+P addition on soil N_2_O flux was stronger than N addition alone.

### Enzyme activities after N and/or P addition

AP and NAG catalyze the hydrolysis and cleavage of molecular bonds in compounds, and are therefore critical for soil P- and N-cycling [44]. In our experiment, AP and NAG activities were respectively increased by 32% and 26% in the N addition plot over the control. This result was not completely consistent with the theory of resource allocation [20], which predicts that when a nutrient is limited, enzyme production increase and vice versa. A significant promotion of N addition on P- and N-cycling enzymes has been found in some terrestrial ecosystems [20, 22, 45]. The way N addition affects AP and NAG may depend on the type of available substrate [46]. In the N-limited ecosystems, one reasonable explanation is that N addition not only increased the plant and microbial biomass in soil (Tables 2 and 4), enhancing the demand for N, and increasing the activities of NAG, but that it also led to a decrease in the available P content (Table 2). A soil feedback control mechanism can increase the activities of soil AP, prompting soil microorganisms to secrete more AP to increase soil available P content [47–49].

A negligible effect of P addition on AP possible was that the short experimental time we used might have prevented us from observing statistically significant changes in AP after three years of P addition [20]. P addition stimulated aboveground biomass and increased the uptake of inorganic N in soil, reducing soil inorganic N content (Table 2), and eventually leaded to an increase in activities of NAG. Nevertheless, our study showed that NAG activities associated with P and N+P addition in comparison with control increased by 33% and 27%, respectively, suggesting that P is more important than inorganic N for regulating the activities of NAG.

## Conclusion

This 3-year field study measured the response of soil N_2_O fluxes and enzyme activities to N and/or P addition in an alpine wetland on the Tibetan Plateau, China. Our study found that cumulative N_2_O flux in the first year of the N+P addition showed a more pronounced response to N addition, which may be a synergic effect of N+P addition on N_2_O flux. But this synergic effect weakened in the following two years, and so our findings emphasize the importance of long-term research when investigating N_2_O flux. N+P addition increased NAG activities by 27%, while it exerted a neutral effect on AP. Our findings could be conductive to understand soil N_2_O fluxes under different nutrient elements conditions, and provide the useful information for improving soil N losses.

## Supporting information

S1 Fig Water filled pore space (WFPS), air temperature (Ta) and soil temperature (Ts) during the growing season from 2014 to 2016 in control plot. (TIF)

S1 Table Correlation coefficients between soil acid phosphatase (AP), β-1, 4-*N*-acetyl-glucosaminnidase (NAG) and soil N_2_O flux under N and/or P addition. (DOCX)

## References

[1] Fang HJ, Cheng SL, Yu GR, Wang YS, Xu MJ, Dang XS, et al Microbial mechanisms responsible for the effects of atmospheric nitrogen deposition on methane uptake and nitrous oxide emission in forest soils: a review. Acta Ecol Sin. 2014; 34(17):4799–4806.

[2] Wang F, Li J, Wang X, Zhang W, Zou B, Neher DA, et al Nitrogen and phosphorus addition impact soil N_2_O emission in a secondary tropical forest of South China. Sci Rep. 2014; 4:5615 10.1038/srep05615 25001013 PMC4085593

[3] Bouwman AF, Beusen AHW, Griffioen J, Groenigen JWV, Hefting MM, Oenema O, et al Global trends and uncertainties in terrestrial denitrification and N_2_O emissions. Philos Trans R Soc London. 2013; 368(1621):91–97. 10.1098/rstb.2013.0112 PMC3682736 23713114

[4] Galloway JN, Townsend AR, Erisman JW, Bekunda M, Cai Z, Freney JR, et al Transformation of the nitrogen cycle: recent trends, questions, and potential solutions. Science. 2008; 320(5878):889–892. 10.1126/science.1136674 18487183

[5] Liu X, Zhang Y, Han W, Tang A, Shen J, Cui Z, et al Enhanced nitrogen deposition over China. Nature. 2013; 494(7438):459–462. 10.1038/nature11917 23426264

[6] Fisk MC, Ratliff TJ, Goswami S, Yanai RD. Synergistic soil response to nitrogen plus phosphorus fertilization in hardwood forests. Biogeochemistry. 2014; 118(1–3):195–204.

[7] Jiang CM, Yu GR, Fang HJ, Gao GM, Li YN. Short-term effect of increasing nitrogen deposition on CO_2_, CH_4_ and N_2_O fluxes in an alpine meadow on the Qinghai-Tibetan Plateau, China. Atmos Environ. 2010; 44(24):2920–2926.

[8] Lund M, Christensen TR, Mastepanov M, Lindroth A, Ström L. Effects of N and P fertilization on the greenhouse gas exchange in two northern peatlands with contrasting N deposition rates. Biogeosci Discuss. 2009; 6(3):2135–2144.

[9] Lu M, Yang YH, Luo YQ, Fang CM, Zhou XH, Chen JK, et al Responses of ecosystem nitrogen cycle to nitrogen addition: a meta-analysis. New Phytol. 2011; 189(4):1040–1050. 10.1111/j.1469-8137.2010.03563.x 21138438

[10] Mori T, Ohta S, Ishizuka S, Konda R, Wicaksono A, Heriyanto J, et al Effects of phosphorus addition on N_2_O and NO emissions from soils of an Acacia mangium plantation. Soil Sci Plant Nutr. 2010; 56(5):782–788.

[11] Zhang W, Zhu X, Luo YQ, Rafique R, Chen H, Huang J, et al Responses of nitrous oxide emissions to nitrogen and phosphorus additions in two tropical plantations with N-fixing vs. non-N-fixing tree species. Biogeosci Discuss. 2014; 11(1):1413–1442.

[12] Mori T, Ohta S, Ishizuka S, Konda R, Wicaksono A, Heriyanto J, et al Phosphorus application reduces N_2_O emissions from tropical leguminous plantation soil when phosphorus uptake is occurring. Biol Fert Soil. 2014; 50(1):45–51.

[13] Zheng MH, Zhang T, Liu L, Zhu WX, Zhang W, Mo JM. Effects of nitrogen and phosphorus additions on nitrous oxide emission in a nitrogen-rich and two nitrogen-limited tropical forests. Biogeosci Discuss. 2016; 13(11):3503–3517.

[14] Hall SJ, Asner GP, Kitayama K. Substrate, climate, and land use controls over soil N dynamics and N-oxide emissions in Borneo. Biogeochemistry. 2004; 70(1):27–58.

[15] Mori T, Ohta S, Ishizuka S, Konda R, Wicaksono A, Heriyanto J, et al Effects of phosphorus addition with and without ammonium, nitrate, or glucose on N_2_O and NO emissions from soil sampled under Acacia mangium plantation and incubated at 100% of the water-filled pore space. Biol Fert Soils. 2013; 49(1):13–21.

[16] Koch AL. The macroeconomics of bacterial growth. London, Academic Press 1985; 16:1–42.

[17] Bai HY, Han JG, Zhang YP. Correlations between physical, chemical and biological properties and denitrifying enzymes activity and N_2_O flux in soil profiles. Agro-Environ Prot. 2002; 21:193–196.

[18] Jian GH, Zhan BL, Yong LZ, Hong YB, Dong Q. Soil enzyme activities and N_2_O emissions under different land management conditions. Bull Environ Contam Toxicol. 2004; 73(1):205–212. 10.1007/s00128-004-0414-0 15386093

[19] German DP, Weintraub MN, Grandy AS, Lauber CL, Rinkes ZL, Allison SD. Optimization of hydrolytic and oxidative enzyme methods for ecosystem studies. Soil Biol Biochem. 2011; 43(7):1387–1397.

[20] Stevend A, Claudiai C, Kathleenk T. Microbial activity and soil respiration under nitrogen addition in Alaskan boreal forest. Global Change Biol. 2008; 14(5):1156–1168.

[21] Turner BL, Wright SJ. The response of microbial biomass and hydrolytic enzymes to a decade of nitrogen, phosphorus, and potassium addition in a lowland tropical rain forest. Biogeochemistry. 2014; 117(1):115–130.

[22] Jing X, Yang XX, Ren F, Zhou HK, Zhu B, He JS. Neutral effect of nitrogen addition and negative effect of phosphorus addition on topsoil extracellular enzymatic activities in an alpine grassland ecosystem. Appl Soil Ecol. 2016; 107:205–213.

[23] Xing Y, Jiang Q, Li W. Landscape spatial patterns changes of the wetland in Qinghai-Tibet Plateau. Ecol Environ Sci. 2009; 18:1010–1015.

[24] Jia YL, Yu GR, He NP, Zhan XY, Fang HJ, Sheng WP, et al Spatial and decadal variations in inorganic nitrogen wet deposition in China induced by human activity. Sci Rep. 2014; 4:3763 10.1038/srep03763 24441731 PMC3895902

[25] Jiang J, Zong N, Song MH, Shi PL, Ma WL, Fu G, et al Responses of ecosystem respiration and its components to fertilization in an alpine meadow on the Tibetan Plateau. Eur J Soil Biol. 2013; 56:101–106.

[26] Gao JQ, Ouyang H, Xu XL, Zhou CP, Zhang F. Effects of temperature and water saturation on CO_2_ production and nitrogen mineralization in alpine wetland soils. Pedosphere. 2009; 19(1):71–77.

[27] Ding WX, Cai ZC, Wang DX. Preliminary budget of methane emissions from natural wetlands in China. Atmos Environ. 2004; 38(5):751–759.

[28] Tian XF, Hu HW, Ding Q, Song MH, Xu XL, Zheng Y, et al Influence of nitrogen fertilization on soil ammonia oxidizer and denitrifier abundance, microbial biomass, and enzyme activities in an alpine meadow. Biol Fert Soils. 2014; 50(4):703–713.

[29] Zheng Y, Kim YC, Tian XF, Chen L, Yang W, Gao C, et al Differential responses of arbuscular mycorrhizal fungi to nitrogen addition in a near pristine Tibetan alpine meadow. Fems Microbiol Ecol. 2014; 89(3):594–605. 10.1111/1574-6941.12361 24890754

[30] Müller AK, Matson AL, Corre MD, Veldkamp E. Soil N_2_O fluxes along an elevation gradient of tropical montane forests under experimental nitrogen and phosphorus addition. Front Earth Sci. 2015; 3:1–2.

[31] Xu K, Wang CM, Yang XT. Five-year study of the effects of simulated nitrogen deposition levels and forms on soil nitrous oxide emissions from a temperate forest in Northern China. Plos One. 2018; 12(12): e0189831 10.1371/journal.pone.0189831 PMC5734751 29253001

[32] Vance ED, Brookes PC, Jenkinson DS. Microbial biomass measurement in forest soils: the use of the chloroform fumigation-incubation method in strongly acid soils. Soil Biol Biochem. 1987; 19(6):697–702.

[33] Guan G, Tu SX, Yang JC, Zhang JF, Yang L. A field study on effects of nitrogen fertilization modes on nutrient uptake, crop yield and soil biological properties in rice-wheat rotation system. Agr Sci China. 2011; 10(8):12540–1261.

[34] Chen H, Zhu Q, Peng CH, Wu N, Wang YF, Fang XQ, et al The impacts of climate change and human activities on biogeochemical cycles on the Qinghai-Tibetan Plateau. Global Change Biol. 2013; 19(10):2940–2955. 10.1111/gcb.12277 23744573

[35] Chen YP, Chen GC, Ye Y. Coastal vegetation invasion increases greenhouse gas emission from wetland soils but also increases soil carbon accumulation. Sci total environ. 2015; 526(1):19–28. 25918889 10.1016/j.scitotenv.2015.04.077

[36] Liu LL, Greaver TL. A review of nitrogen enrichment effects on three biogenic GHGs: the CO_2_ sink may be largely offset by stimulated N_2_O and CH_4_ emission. Ecol Lett. 2009; 12(10):1103–1117. 10.1111/j.1461-0248.2009.01351.x 19694782

[37] Bai JB, Xu XL, Fu G, Song MH, He YT, Jing J. Effects of temperature and nitrogen input on nitrogen mineralization in alpine soils on Tibetan Plateau. Agr Sci, Tech. 2011; 12(12):1909–1912.

[38] Wang H, Yu L, Zhang Z, Liu W, Chen L, Gao G, et al Molecular mechanisms of water table lowering and nitrogen deposition in affecting greenhouse gas emissions from a Tibetan alpine wetland. Glob Chang Biol. 2017; 23(2):815–829. 10.1111/gcb.13467 27536811

[39] Mori T, Wachrinrat C, Staporn D, Meunpong P, Suebsai W, Matsubara K, et al Effects of phosphorus addition on nitrogen cycle and fluxes of N_2_O and CH_4_ in tropical tree plantation soils in Thailand. Agriculture and Natural Resources. 2017; 51(2):91–95.

[40] Mehnaz K, Keitel C, Dijkstra FA. Effect of carbon and phosphorus addition on microbial respiration, N_2_O emission, and gross nitrogen mineralization in a phosphorus-limited grassland soil. Biol Fert soils. 2018; 54(4):481–493.

[41] Baral BR, Kuyper TW, Van Groenigen JW. Liebig’s law of the minimum applied to a greenhouse gas: alleviation of P-limitation reduces soil N_2_O emission. Plant Soil. 2014; 374:539–548.

[42] Fang YT, Mo JM, Gundersen P, Zhou GY, Li DJ. Nitrogen transformations in forest soils and its responses to atmospheric nitrogen deposition: a literature review. Acta Ecol Sin. 2004; 24(7):1523–1531.

[43] Kadono A, Funakawa S, Kosaki T. Factors controlling mineralization of soil organic matter in the Eurasian steppe. Soil Biol Biochem. 2008; 40(4):947–955.

[44] Acosta-Martínez V, Cruz L, Sotomayor-Ramírez D, Pérez-Alegría. Enzyme activities as affected by soil properties and land use in a tropical watershed. Appl Soil Ecol. 2007; 35(1):35–45.

[45] Wang QK, Wang SL, Liu YX. Responses to N and P fertilization in a young Eucalyptus dunnii plantation: microbial properties, enzyme activities and dissolved organic matter. Appl Soil Ecol. 2008; 40(3):484–490.

[46] Sinsabaugh RL, Lauber CL, Weintraub MN, Ahmed B, Allison SD, Crenshaw C, et al Stoichiometry of soil enzyme activity at global scale. Ecol Lett. 2008; 11(11):1252–1264. 10.1111/j.1461-0248.2008.01245.x 18823393

[47] Chung HG, Zak DR, Reich PB, Ellsworth DS. Plant species richness, elevated CO_2_, and atmospheric nitrogen deposition alter soil microbial community composition and function. Global Change Biol. 2007; 13(5):980–989.

[48] Xia X, Gu J, Gao H, Qin QJ, Liu L, Xie YY. Effect of inorganic fertilizer combined with organic manure on soil hydrolase activities during the growth of corn. Agricultural Research in the Arid Areas. 2010; 28:38–42.

[49] Houlton BZ, Marklein AR. Nitrogen inputs accelerate phosphorus cycling rates across a wide variety of terrestrial ecosystems. New Phytol. 2012; 193:696–704. 10.1111/j.1469-8137.2011.03967.x 22122515

