# Supplementary material for: Contrasting effects of nitrogen and phosphorus additions on soil nitrous oxide fluxes and enzyme activities in an alpine wetland of the Tibetan Plateau
Source: PLoS One. 2019 May 2;14(5):e0216244. doi: 10.1371/journal.pone.0216244 (PMC6497268; doi:10.1371/journal.pone.0216244)
Supplement: S1 Table — (DOCX) [file pone.0216244.s002.docx]

**S1 Table. Correlation coefficients between soil acid phosphatase (AP), β-1, 4-*N*-acetyl-glucosaminnidase (NAG) and soil N_2_O flux under N and/or P addition.**

|  | **Soil N_2_O flux** | | | |
| --- | --- | --- | --- | --- |
|  | Control | N | P | N+P |
| **AP** | 0.052 | 0.197 | 0.026 | 0.136 |
| **NAG** | 0.748** | 0.697** | 0.798** | 0.632** |

Pearson correlation coefficients (r) and their significance (*P*) are given as: ***P* < 0.01.
